# Zein Nanoparticles Improve the Oral Bioavailability of Curcumin in Wistar Rats

**DOI:** 10.3390/pharmaceutics13030361

**Published:** 2021-03-09

**Authors:** Ana Brotons-Canto, Carlos J. González-Navarro, Ana Gloria Gil, Eduardo Asin-Prieto, María José Saiz, Josep Manuel Llabrés

**Affiliations:** 1 Nucaps Nanotechnology S.L., 31110 Noain, Spain; 2 Center for Nutrition Research, University of Navarra, 31008 Pamplona, Spain; cgnavarro@unav.es; 3 Drug Development Unit, Department of Pharmacology and Toxicology, Faculty of Pharmacy and Nutrition, University of Navarra, 31008 Pamplona, Spain; agil@unav.es; 4 Inserm U1070, Pôle Biologie Santé, Université de Poitiers, 86000 Poitiers, France; eduardo.asin@inserm.fr; 5 National Center for Food Technology and Safety, CNTA, 31570 San Adrian, Spain; mjsaiz@cnta.es; 6 Plantas Medicinales y Complementos Alimenticios, S.A. (PLAMECA), 08780 Palleja, Spain; jmllabres@plameca.com

**Keywords:** antioxidant, bioavailability, curcumin, curcuminoid, nanoparticles

## Abstract

Curcumin is a natural compound obtained from turmeric root with high antioxidant and anti-inflammatory activities. However, clinical application of curcumin has been limited due to its low solubility and bioavailability and rapid metabolism and degradation. This study was conducted to evaluate the effect of curcumin incorporation in zein nanoparticles on the pharmacokinetic parameters of systemic curcumin in plasma. Wistar rats were administered a single oral dose of 250 mg/kg of standard curcumin (control) or nanocurcumin (zein-based nanoparticles, Nucaps). The proposed new formulation was also compared with two commercially available curcumin complexes. Blood samples were collected at different times, and plasma levels were determined using HPLC-MS/MS. Overall, nanocurcumin (Nucaps) formulation was well tolerated and showed a 9-fold increase in oral bioavailability when compared to the standard curcumin natural extract. In addition, the nanoparticles prepared in this study demonstrated a bioavailability profile superior to that of other bioavailability-enhanced curcumin complexes currently available in the marketplace. Thus, our nanoparticle-based formulation has shown great potential as a nutraceutical for the oral administration of curcumin.

## 1. Introduction

*Curcuma longa*, commonly known as turmeric, is a plant member of the ginger family (*Zingiberaceae*). This plant is endemic and prevalent in tropical and sub-tropical regions [1], and it is widely cultivated in India and China [2].

The rhizome of *Curcuma longa* contains carbohydrates (60–70%), proteins (6–8%), essential (3–7%) and fixed oils (5–10%), fiber (2–7%), minerals (3–7%) and curcuminoids (2–6%) [3]. The latter is a mixture of curcumin (CC) and two derivatives: demethoxy-curcumin (DMC) and bis-demethoxy-curcumin (BDMC) [4]. The relative proportion of the three curcuminoids usually remains stable (ca. 70% CC, 17% DMC, and 3% BDMC), although it might present slight variations with cultivar [5].

Although CC is the most abundant and widely studied compound, both DMC and BDMC have been reported to elicit a similar biological activity to that of CC [6]. Turmeric, and specifically CC, has been widely used as a safe remedy, spice, and dye since 1900 B.C. [6,7,8,9]. In fact, curcuminoids have been consumed as therapeutic infusions worldwide over centuries [1] with different purposes. Ayurvedic medicine uses turmeric for the treatment of different ailments and symptoms involved in gynecological, gastric, hepatic, hematological, and biliary disorders; infectious diseases (coryza, cough, etc.) [2]; skin disorders, such as acne, psoriasis or dermatitis; anorexia; rheumatism; and sinusitis [10]. Chinese traditional medicine has used CC for the therapeutics of diseases associated with abdominal pain [11], whereas other oriental cultures used it as an anti-inflammatory, antioxidant, anti-carcinogenic. and antimicrobial reagent [1].

These benefits have been studied in vitro and in vivo, proving and reporting the antioxidant [12,13,14,15], anti-inflammatory [16,17], and antibacterial [18] activity of CC. In fact, the analgesic effect of curcuminoids has been demonstrated in osteoarthritis among other pathologies [19]. In a randomized controlled study, the use of curcuma showed an ability to alleviate knee pain comparable to that of ibuprofen [20]. In another randomized, double-blinded placebo-controlled study, the use of CC reduced knee pain and celecoxib administration dependence when compared with the placebo [21].

At the molecular level, CC is a pleiotropic molecule that is able to interact with a large number of molecular targets, such as proteins, enzymes, DNA, RNA, and carrier molecules. This provides CC with the ability to exert various biological activities, either directly binding the receptors or indirectly through up- or down-regulation mechanisms [3]. In fact, CC suppresses NF-κB activation [3], and down-regulates the expression of genes involved in cell apoptosis, proliferation, and transformation [22]. It also suppresses the activity of enzymes, including COX-2 [23], protein kinases [24], and cytokines, and growth factors involved in the inflammatory response, such as interleukins (IL) 1, 2, 6, 8, and 12; fibroblast growth factor-2; and TNFα [25], which can be related to its anti-inflammatory and antioxidant capacities [26].

Despite the demonstrated potential activity of turmeric, its health benefits are hindered by its lack of water solubility (11 ng/mL in aqueous buffer, pH 5) [9,27] and the low bioavailability [28] showed when orally administered caused by a poor absorption, high rate of first-pass metabolism, and rapid systemic elimination [8]. In fact, the oral bioavailability of CC has been estimated to be ca. 1% [29]. As a result, different strategies are being studied in order to overcome the low solubility and low bioavailability. In this regard, some efforts have explored the concomitant administration of CC and either metabolism inhibitors (e.g., piperine) [8] or compounds with a synergistic effect (e.g., quercetin [30], genistein [31], and resveratrol [32]).

In addition, the preparation of nanoformulations (micelles, liposomes, protein-based nanoparticles, etc.) focuses on the increase in the solubility of the active ingredients and the protection against degradation through the gastrointestinal tract [33,34,35,36]. However, the adequacy for human use of the ingredients in this kind of formulation and their techno-economic feasibility are not yet clear [26,36,37]. In this context, the use of GRAS (generally recognized as safe) substances, with an easy preparative process in absence of toxic cross-linking agents, is desirable. Among others, zein (corn protein) is currently the subject of great interest due to its high coating capacity, biocompatibility, and biodegradability, which makes it an excellent nanocarrier for oral drug delivery [38,39].

In this context, the aims of this work were to prepare, characterize, and evaluate a curcuminoid-loaded nanoparticulate formulation based on zein and to study in vivo its oral bioavailability and pharmacokinetic properties in rats.

## 2. Materials and Methods

### 2.1. Chemicals

Zein was obtained from FloChemical Corporation (Ashburnham, MA, USA). Curcuminoid extract 95%, Curarti^®^, a beta-cyclodextrin complex of curcuminoids, and a curcumin–phosphatidylcholine complex (CPC) were supplied by Plameca (Pallejà, Spain). Acetic acid, acetonitrile (ACN), dimethyl sulfoxide (DMSO), di-sodium hydrogen phosphate anhydrous, ethyl acetate, formic acid (FA), phosphoric acid, potassium chloride, sodium acetate, and sodium chloride were purchased from Scharlau Chemie (Sentmenat, Spain). β-Glucuronidase from bovine liver, potassium di-hydrogen phosphate, and sulfatase from *Helix pomatia* were purchased from Sigma-Aldrich (Darmstadt, Germany). Standards for curcumin, curcumin-d6, and (2E)-demethoxycurcumin-d7 were purchased from Toronto Research Chemicals (Toronto, ON, Canada). L-lysine and absolute ethanol were acquired from Panreac (Barcelona, Spain). Ultrapure water was obtained from a Milli-Q water purification system (Bedford, MA, USA). Mannitol was from Merck (Darmstadt, Germany). Ethanol 70% (v/v) was purchased from VWR Chemicals (Barcelona, Spain).

### 2.2. Optimization of Curcumin-to-Zein Ratio for Zein Nanoparticles

Zein nanoparticles were prepared following a method previously described [38,39]. Briefly, 100 mg of zein and 15 mg of lysine were dissolved in ethanol (70% *v/v*) and incubated with different mass ratios of curcuminoid extracts (in order to reach final concentrations of 6, 10, and 15% of curcuminoids) under magnetic stirring at room temperature. Then, nanoparticles were formed by the addition of milli-Q water until induction of the desolvation process. Afterwards, a mannitol solution (100 mg/mL) was added to the already formed nanoparticles prior to the drying step in a Büchi Mini Spray Drier B-290 apparatus (Büchi Labortechnik AG, Flawil, Switzerland) under the following experimental conditions: inlet temperature of 100 °C, outlet temperature of 60–70 °C, air pressure at 4–5 bar, pumping rate of 10–12 mL/min, aspirator at 80%, and air flow set at 900 mL/h.

According to the final concentration of curcuminoids (6, 10, and 15%), the resulting nanoparticles were identified as NPZ-CC6, NPZ-CC10, and NPZ-CC15, respectively.

In addition, empty zein nanoparticles were prepared in the same way as described above but in the absence of curcuminoid extract. These nanoparticles were identified as NPZ.

### 2.3. Physicochemical Characterization of Curcuminoid-Loaded Zein Nanoparticles

#### 2.3.1. Mean Size, Polydispersity Index, Zeta Potential, and Process Yield

The particle size, polydispersity index (PDI) and zeta-potential were determined by photon correlation spectroscopy and electrophoretic laser Doppler anemometry, respectively, using a Zetasizer analyzer system (Brookhaven Instruments Corporation, Holtsville, NY, USA). The diameter and PDI of the nanoparticles were determined after dispersion in ultrapure water (1/10) and measured at 25 °C by dynamic light scattering angle of 90°. The zeta potential was determined after dilution of 2 mg of the sample in 2 mL of ultrapure water.

#### 2.3.2. Curcuminoid Quantification

Curcuminoid quantification was determined by reverse-phase high performance liquid chromatography (HPLC) with UV detection. The chromatographic separation was performed using a liquid chromatographic system equipped with an Alliance 2695 system connected to a Waters 2998 photodiode array detector (Waters Corp., Mildford, MA, USA). Detection was carried out at 427 nm for CC, 422 nm for DMC, and 417 nm for BDMC.

Chromatographic separation was achieved using a Sunfire C18, 5 μm (150 × 4.6 mm) column (Waters Corp., Mildford, MA, USA). The column temperature was 25 °C, and samples were stored at 10 °C before injection. Injection volume was set at 20 μL.

The isocratic mobile phase was 1% (*v/v*) acetic acid in HPLC-grade water (A) and ACN (B) (1:1), at a flow rate of 1.0 mL/min with a total run time of 10 min. The retention time for each curcuminoid was 6.5, 7.1, and 7.8 min for BDMC, DMC, and CC, respectively.

For the quantification of curcuminoids in the nanoparticles, 10 mg of the formulation was dispersed in 1 mL of water and centrifuged at 30,500 g for 20 min. Then, the pellet was dissolved in 10 mL ethanol 70% and shaken mechanically until complete dissolution.

Each sample was filtered through a 0.45 µm PVDF filter and injected in the HPLC system for its quantification. Each sample was analyzed in triplicate, and the results are expressed as the amount of each curcuminoid (in μg) per mg of formulation.

Afterwards, the encapsulation efficiency (E.E.) was calculated by the following Equation (1):

EE (%) = (AcurTQ/AcurT) × 100(1)

where AcurTQ is the total amount of curcuminoids quantified in the formulation, and AcurT is the targeted amount of the curcuminoids employed in the preparation of nanoparticles.

### 2.4. In Vivo Pharmacokinetic Study in Male Wistar Rats

#### 2.4.1. Ethical Statement

All animal procedures were performed in accordance with the national and institutional Guidelines for Care and Use of Laboratory Animals, with the consent of the Food Safety and Environmental Health Service of the Government of Navarra, Spain. The protocol was approved by the Ethics Committee for Animal Experimentation of the University of Navarra (protocol reference CEEA/009-15).

#### 2.4.2. In Vivo Study

The selected optimum formula (identified as NPZ-CC from now) was subjected to pharmacokinetic study in the plasma of male Wistar rats (Envigo Research Models and Services, Gannat, France) in comparison with free curcuminoids. For comparative purposes, two commercially available modified formulations of curcuminoids were also tested: Curarti^®^, a beta-cyclodextrin-complex of curcuminoids, and a CPC.

Male Wistar rats weighing 240 g (CV 20%) were divided evenly into 4 groups (*n* = 6), with each group administered either the selected formulation (NPZ-CC), free curcuminoids, CPC, or Curarti^®^ complex as a single dose of 250 mg/kg by oral route.

Animals from each group were subjected to blood sampling at 0 (pre-administration) 0.25, 0.5, 1, 1.5, 2, 4, 6, and 24 h after administration. Blood samples (1 mL) were withdrawn from the retro-orbital plexus and collected in lithium heparin plasma tubes. Samples were immediately centrifuged at 2000 g for 10 min at 4 °C. The supernatant (plasma) was transferred to clean tubes and kept frozen at −80 °C until analysis.

#### 2.4.3. Quantification of Curcuminoids in Plasma by HPLC-MS/MS

The concentrations of the three free curcuminoids (CC, DMC and BDMC) and the total curcuminoid concentrations in rat plasma were determined at Kymos Pharma Services S.L. (Cerdanyola del Valles, Spain) by an LC-MS/MS qualified method according to the guidelines of CDER Industry [40], the AAPSJ workshop report [41], and the Guideline on bioanalytical method validation [42], as described below.

In order to determine the total curcuminoid concentration, enzymatic hydrolysis was previously performed with β-glucuronidase from bovine liver and sulfatase from *Helix pomatia* was performed [43].

The chromatographic system was equipped with a Luna^®^ C18 column (4.6 × 50 mm; particle size: 5 µm) (Phenomenex Inc., Torrance, CA, USA). The column was conditioned at 10°C, and the injection volume was set at 20 μL. The mobile phase, pumped at 0.8 mL/min, was a gradient mixture (Table 1) of water (A, 0.1% FA) and ACN (B, 0.1% FA). Under these conditions, the total run-time was set at 7 min.

Sample quantification was carried out by a mass spectrometer API4000 with positive electrospray ionization using a TurboIonspray probe and Analyst^®^ software (version 1.7) for signal processing. Table 2 details the conditions and characteristics of the MS/MS system.

#### 2.4.4. Pharmacokinetic Analysis

Non-compartmental pharmacokinetic analysis for the different curcuminoids per formulation was performed using MS Excel add-in program and PK solver [44], and graphical representation was performed using GraphPad Prism v6 (GraphPad Software, San Diego, CA, USA).

The following pharmacokinetic-related metrics were estimated for each formulation and analyzed compound: maximum plasma concentration or peak concentration (Cmax) and the time at which the Cmax was reached (Tmax), which provided insight into the extent and rate of the absorption process; area under the plasma concentration–time curve from time 0 to last observation (AUC_0-tlast_) and area under the plasma concentration–time curve from time 0 to infinity (AUC_0-∞_), which provided insight into the partial and total exposure to the curcuminoids. Furthermore, the relative oral bioavailability (Fr) for the different curcuminoids was estimated using the following equation:

Fr = (AUC_0-∞,test_/D_test_)/(AUC_0-∞,ref_/D_ref_) (2)

where AUC_0-∞,test_ and AUC_0-∞,ref_ represent the area under the plasma concentration–time curve from time 0 to infinity for the different modified test formulations (CPC, Curarti^®^ and NPZ-CC) and the free curcuminoids, respectively. For Fr estimation, the AUC_0-∞,test_ and AUC_0-∞,ref_ were normalized by the actual dose of each curcuminoid administered per formulation test and reference (D_test_ and D_ref_, respectively).

### 2.5. Statistical Analysis

The physicochemical characteristics of nanoparticles as well as pharmacokinetic metrics were compared using the comparison of means tests by Student’s t test. Differences providing a *p*-value lower than 0.05 were considered statistically significant. All statistical analyses were performed using GraphPad Prism v6 (GraphPad Software, San Diego, CA, USA).

## 3. Results

### 3.1. Optimization of the Preparation Process of Curcuminoid-Loaded Nanoparticles

In order to evaluate the impact of the curcuminoid extract in the physicochemical characteristics of the nanoparticles, different curcuminoids-to-zein ratios were evaluated and compared with respect to NPZ. The resulting nanoparticles had theoretical curcuminoid concentration of 6, 10, and 15%. Table 3 summarizes the main results of the nanoparticles characteristics for optimization. In the case of NPZ, the mean size of the formulation was about 200 nm. Particles with a theoretical concentrations of 6 and 10% of curcuminoids (real curcuminoid concentration of 5.20% and 9.18%, respectively) displayed similar results (192 and 222 nm, respectively). In all cases, the PDI of the formulations remained under 0.3 reflecting homogeneous systems. However, higher payloads (e.g., 15% theoretical, corresponding with the 9.70% real concentration) increased the particle size to 763 nm (Table 3) and PDI to around 0.3.

### 3.2. Nanoparticle Characterization

Based on the optimization study, NPZ-CC-10 was selected to continue with the experiment, as it has the highest curcuminoid load without significantly affecting neither particle size nor PDI. For simplicity, the selected formulation (NPZ-CC-10) is referred to as NPZ-CC in the remainder of the manuscript.

Table 4 summarizes the main physicochemical characteristics of these nanoparticles. The mean sizes of NPZ and NPZ-CC were similar, with minor statistically significant differences between formulations (232 vs. 222 nm, *p* < 0.05). These differences are associated with the encapsulation process, which modifies the spatial arrangement of zein amino acid chains to accommodate curcuminoid molecules inside the fractal-like aggregates of the zein nanoparticle structure [45]. In both cases, the preparation process resulted into homogeneous batches of nanoparticles (PDI < 0.3, *n* = 3). The total curcuminoid loading was calculated to be 9.18%, with an E.E. close to 98%.

The mean size (nm) and PDI of the commercialized curcumins CPC and Curarti^®^ were measure as control. CPC had a mean size of 1257 ± 1189 and high PDI (PDI value 1), whereas Curarti^®^ showed a mean size of 3217 ± 2163 and a PDI value of 0.683 ± 0.275.

### 3.3. In Vivo Study

The pharmacokinetic behavior of the optimum formula was evaluated in rats and compared with the two commercially available formulations and the free curcuminoids. Wistar rats (250 g) were divided into four groups (*n* = 6 rats/group). Each group of animals received a single oral dose of 250 mg/kg of total curcuminoids either free (curcuminoid extract) in suspension or formulated in different systems: as a complex (Curarti^®^), as a lipid-based nanoparticle (CPC), or the zein-based formulation developed by Nucaps (NPZ-CC). Table 5 shows the percentage of each component present in the formulations.

The percentage of curcuminoids in the formulations (Table 5) was used to correct the pharmacokinetic estimations in order to ensure the comparability among the different treatment groups.

The concentration profile of each curcuminoid over time was represented and evaluated, and the pharmacokinetic variables were estimated using PKSolver (Pharsight, Mountain View, CA, USA) [44]. The Fr of CC was calculated for each test formulation (Curarti^®^, CPC and NPZ-CC).

**Figure 1:**
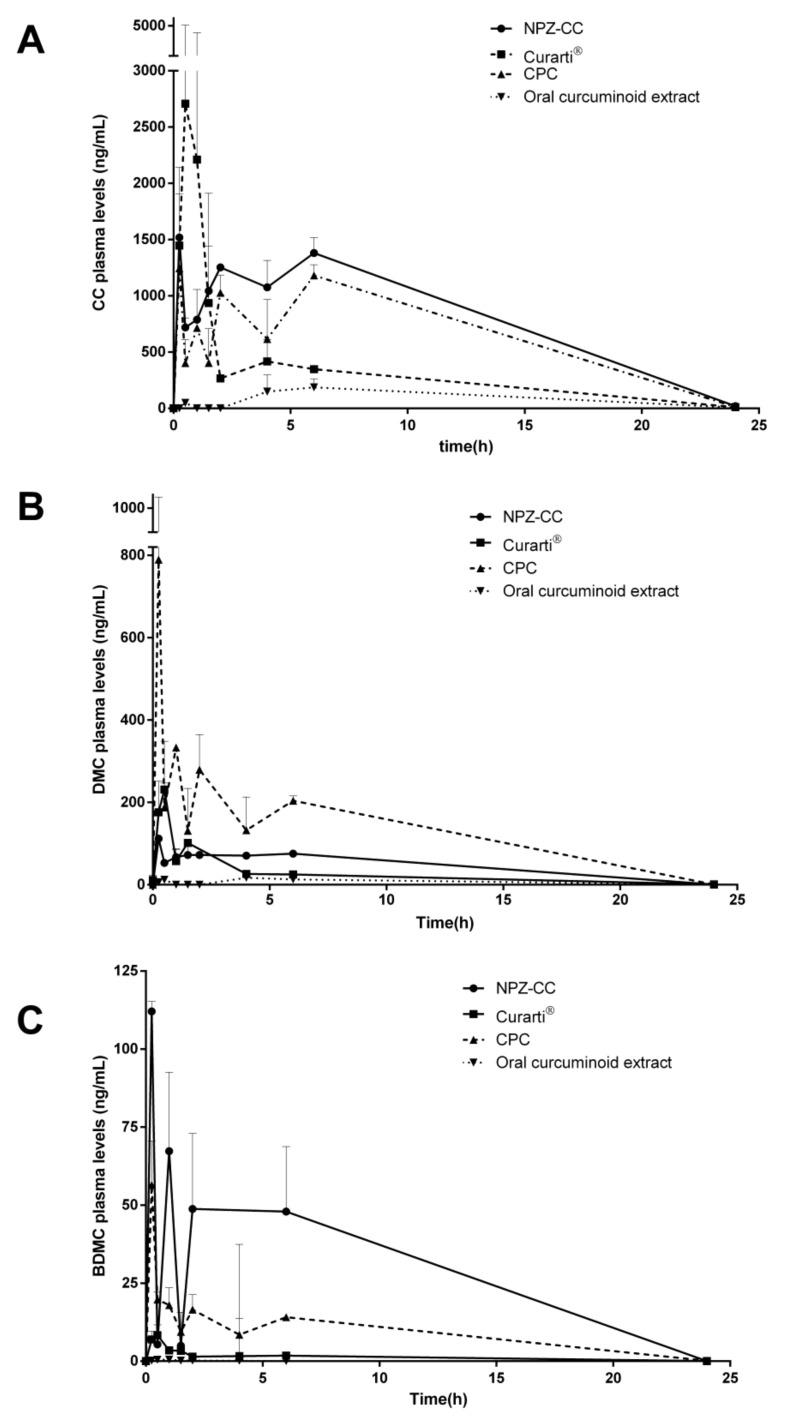
Representation of the CC (**A**), DMC (**B**), and BDMC (**C**) plasma concentrations vs. time after the oral administration of a single dose of curcuminoids. Curcuminoid administration was performed either as free extract of curcuminoids (reference formulation, ▼, dotted line) or entrapped into three formulated systems: Nucaps developed formulation (NPZ-CC, ●, solid line) and two commercial formulations, a lecithin-based nanoparticle formulation (CPC, ▲, dashed line) and a cyclodextrin complex formulation (Curarti^®^, ■, solid line).

Overall, noticeable differences in the average plasma concentrations are shown depending on the administered formulation and the analyzed compound (Figure 1).

Regarding CC, the oral administration of the free curcuminoids reached a peak concentration (Cmax) value of 186 ng/mL in plasma at 6 h. In the case of the oral administration of the beta-cyclodextrin complexed curcuminoids (Curarti^®^) as an aqueous solution, the CC levels in the plasma of animals increased rapidly after administration during the first hour post-administration, showing a high absorption rate and reaching the Cmax at 0.5 h. Then, the amount of CC decreased rapidly until the end of the experiment (24 h post-administration).

For nanoparticle formulations (CPC and NPZ-CC), the CC plasma levels vs. time displayed similar profiles with a first peak within the first hour post-administration and a second phase with a slow decrease. However, in all cases, the plasma levels of CC obtained with NPZ-CC were higher than those observed for the oral administration of CPC (Figure 1A). The absorption rate of CC estimated as Tmax was similar to those obtained by Cuomo, et al. [46]. Similarly, the Tmax of CPC was reduced when compared to that of the free form [46].

The Fr reached after the oral administration of the formulations clearly shows that the administration of CC into NPZ-CC, Curarti^®^, and CPC improves the curcuminoid exposure in comparison with the free extract. In the case of Curarti^®^, a 3-fold increase in bioavailability was obtained, whereas NPZ-CC and CPC formulations produced a 7.5- and 6.1-fold increase in exposure, respectively, compared with that of the administration of the free curcuminoid (Table 6).

Regarding DMC, Figure 1B shows the plasma concentration levels of DMC after oral administration of curcuminoids as a single dose to rats. When administered as a suspension (free extract), a high rate of non-detectable levels for DMC was observed in short time periods, indicating a very low absorption profile, and, as a consequence, a low Cmax was obtained over a long time period (17 ng/mL at Tmax equal to 4 h) when compared to the other formulations. On the other hand, when administered as a beta-cyclodextrin complexed formulation (Curarti^®^), the DMC plasma levels displayed a Cmax of around 231 ng/mL at 30 min after administration and rapid decrease afterwards (Table 5). In the case of the nanoparticle-based formulation (NPZ-CC and CPC), the plasma levels reached the Cmax at 0.25 h. In the case of CPC formulation, the DMC profile reached higher levels in comparison with the other formulations, showing an up-and-down plasma concentration profile during the first 6 h. This behavior was also shown for CC (Figure 1A).

The exposure values (AUC_0-tlast_) obtained for the test formulations were higher than that for free DMC, especially in the nanoparticle-based formulations, as mentioned above. Similarly, the peak plasma concentration (Cmax) of DMC in the tested formulations was between 6 and 46 times higher than that of the free curcuminoid extract. However, no statistically significant differences were found, probably due to the high variability observed in the Curarti^®^ and CPC groups. Finally, in the analysis of the Fr of DMC, when incorporated into zein nanoparticles (NPZ-CC), the exposure was around 6 times higher than that obtained after oral administration of the free curcuminoids but almost three times lower than the DMC exposure obtained after CPC administration.

Figure 1C represents the BDMC levels after the administration of curcuminoids in different formulations. As shown for CC and DMC, BDMC also presents a highly variable pharmacokinetic profile depending on the type of formulation used for its administration. Nanoparticle-based formulations achieved higher levels of BDMC, showing an initial peak and a moderate decrease in the levels over time. On the contrary, the Curarti^®^ formulation elicits an initial peak followed by a subsequent dramatic decrease in BDMC levels. These patterns are consistent with the results previously shown for CC and DMC.

Finally, Table 5 also summarizes the main pharmacokinetic variables estimated for the curcuminoid BDMC with a non-compartmental analysis of the experimental data obtained after the administration of the different formulations. The AUC values for BDMC from the modified formulation (ranging from 15 to 215 ng·h/mL) were higher than those observed for the curcuminoid suspension (1.1 ng·h/mL for free curcuminoids). Accordingly, the Fr of BDMC when incorporated into the modified formulations NPZ-CC, Curarti^®^, and CPC was calculated to be between 20 and 548 times higher than that for the free curcuminoid administration.

Taking this into account, it is clear that the formulation of curcuminoids enhances their bioavailability after oral administration compared to the free extract. Additionally, differences in terms of extent and pattern were detected among the modified formulations, whilst the formulations in nanoparticle-based systems (CPC or NPZ-CC) were able to modify the pattern of release/absorption, pointing to a bimodal profile and a consistently higher bioavailability for all the curcuminoids with respect to the non-nanoparticle formulation evaluated in this study. Besides, the fluctuations shown in the pharmacokinetic profiles might be indicative of the presence of additional mechanisms affecting the kinetics of the compound, as can be observed in the case of first-pass metabolism and enterohepatic circulation [47].

## 4. Discussion

Curcuminoids have been widely used in order to mitigate different ailments for centuries. In recent years, curcumin has been extensively researched, and only in the last century have important functions of curcumin been revealed [1,3]. In fact, various preclinical studies performed in cell culture and animal models showed the antiproliferative, anti-invasive, and antiangiogenic ability of curcumin [13,23,24,25,26,27,28,48]. Moreover, recent clinical trials with positive results in different ailments and diseases, such as cancer, osteoarthritis, or knee pain [21,22,23,24,25,29], have strengthened the interest in this compound.

Nevertheless, due to its low water solubility and oral bioavailability, its use is limited. Nanoencapsulation has been shown in the past to be a promising strategy to overcome these limitations. Previous works evaluated the potential of different formulations for the oral administration of CC. All these efforts show the potential for the use of drug delivery systems to improve the exposure to orally administered curcuminoids [35,48,49,50]. Nevertheless, most of the investigations addressing the preparation of nanoparticle-based systems use toxic solvents, such as acetone or methanol, which makes these techniques not applicable to the food industry [51,52].

Therefore, in this work, a new zein-based non-toxic curcuminoid formulation was optimized, and its oral bioavailability was evaluated.

It is important to highlight that the mean size of both formulations is higher than 100 nm, and, therefore, they are not considered nanomaterials according to the definition provided by the European Commission [53], and they are not subjected to nanomaterial restrictions.

Overall, the results of our research are consistent with previous studies of different strategies to increase the bioavailability of CC, including the oral administration of CPC [54]. In fact, the absorption rate of CC was similar to that obtained by Cuomo, et al. as shown by Tmax [46].

In this case, the increase in the bioavailability of CC in such lecithin complexes has been attributed to the impact of the digested complexes [54,55,56]. These complexes can be converted into micelles and vesicles of phospholipids and bile salts of the intestinal fluid, promoting an increase in the CC solubilization capacity due to the increase in the number of non-polar domains available to incorporate hydrophobic bioactivity. Thus, more CC molecules could be solubilized [57]. Similarly, the high compatibility of cyclodextrins and curcumin, as in the case of the Curarti^®^ complex, may increase the stability of CC in aqueous mediums and, therefore, contribute to its absorption [49,58].

In the case of zein nanoparticles, this enhancement has been associated with an increase in the water solubility of CC by a mechanism not well understood [55], along with the mucoadhesive properties of zein nanoparticles [59] and the subsequent capacity to increase the residence time of the active ingredient at the site of absorption [59]. This increase in bioavailability has been demonstrated to increase the efficacy of the active ingredient for different substances, such as resveratrol and glibenclamide [38,45]. In the case of resveratrol, the incorporation of the active ingredient into zein nanoparticles and the increase in the bioavailability of it has been demonstrated to improve the anti-inflammatory activity of resveratrol in a mice model of endotoxic shock [38]. Similar results were obtained with other substances, such as quercetin [60,61].

An important feature of this study with NPZ-CC formulation compared with other nanoparticle-based systems is the co-encapsulation of the three curcuminoids (CC, DMC, and BDMC). In the literature, different studies have demonstrated an improvement in the pharmacokinetic properties of CC from synthetic sources [35,62,63]. Despite the fact that the pharmacological benefits of CC have been documented extensively, it is important to highlight that DMC and BDMC also possess promising pharmacological effects [64]. Therefore, obtaining a single formulation able to improve the pharmacokinetic profile of all the three major curcuminoids could result in the achievement of a greater therapeutic benefit [34]. Additionally, the Fr of CC was higher for NPZ-CC than for the other two commercial products (CPC and Curarti^®^).

Finally, it is important to point out that the limited sampling per animal along with the high variability detected may represent a limitation in terms of statistical analysis. As a result, statistically significant differences were not detected in most cases, despite the fact that the magnitude of the effect size is considerable. In this sense, additional studies are needed in order to confirm the observations of this study. Nevertheless, the results obtained agree with previous works as discussed above. All these results show the potential use of zein nanoparticles to formulate curcuminoids in order to enhance their pharmacokinetic properties.

## 5. Conclusions

In this work, zein nanoparticles containing the three natural curcuminoids (NPZ-CC) CC, BDMC, and DMC were satisfactorily prepared and optimized. NPZ-CC exhibited high E.E. (98%) and loading capacity (9.18%). These particles have a preparation procedure that only requires natural non-toxic ingredients and are not subjected to nanomaterial restrictions. This nanoparticle-based system modifies the pattern of release/absorption with additional mechanisms affecting the kinetics of curcuminoids and thus, their bioavailability. In all cases, the plasma levels of curcuminoids obtained with NPZ-CC were higher than those observed for the oral administration of other formulations (showing a 9.17-fold increase in bioavailability in comparison to standard free curcuminoid extracts). Therefore, NPZ-CC is able to improve the three major curcuminoids’ absorption profiles.

## Figures and Tables

**Table 1:** LC-MS/MS gradient for curcuminoid quantification in nanoparticles. A: water (0.1% formic acid (FA)) and B: acetonitrile (ACN; 0.1% FA).

Time (min)	%A (H2O 0.1% FA)	%B (ACN 0.1% FA)
0.0	50	50
4.0	50	50
4.1	10	90
5.5	10	90
5.6	50	50
7.0	50	50

**Table 2:** Operational parameters of the MS/MS detector. MRM: multiple reaction monitoring; Gas 1: nebulizer; Gas 2: turbo ion; CUR: curtain gas; CAD: collision gas; IS: ion spray voltage; TEM: source temperature.

Operational Parameter	Value
Ionization mode	Turbo IonsSpray (TIS)
Scan type	Positive MRM
Gas 1	50 psi
Gas 2	60 psi
CUR	40
CAD	6
IS	5000 V
TEM	550 °C
Duration	7 min

**Table 3:** Influence of the curcuminoid encapsulation on the physicochemical properties of the resulting nanoparticles. PDI: Polydispersity Index, NPZ: empty zein nanoparticles, NPZ-CC: curcuminoid-loaded zein nanoparticles Data are expressed as mean ± SD (*n* = 3).

Formulation	Mean Size (nm)	PDI	Total Curcuminoid Content (%)
NPZ	232 ± 3	0.113 ± 0.023	-
NPZ-CC-6	192 ± 7	0.052 ± 0.055	5.20
NPZ-CC-10	222 ± 3	0.230 ± 0.08	9.18
NPZ-CC-15	763 ± 106	0.307 ± 0.052	9.70

**Table 4:** Physicochemical characteristics of NPZ and curcuminoid-loaded zein nanoparticles (NPZ-CC). Mean size, PDI, and Z potential expressed as mean ± SD (*n* = 3). BDMC: Bisdemethoxycurcumin, DMC: demethoxycurcumin. Statistical differences are represented as asterisk: *p* < 0.05 (*) and *p* < 0.01 (**).

Formulation	Mean Size (nm)	PDI	Z Potential (mV)	CC	BDMC	DMC	Total Curcuminoids
NPZ	232 ± 3	0.113 ± 0.023	−43 ± 7	-	-	-	-
NPZ-CC^®^	222 ± 3 *	0.230 ± 0.008 **	−47 ± 1	7.29	0.48	1.41	9.18

**Table 5:** Percentage of each curcuminoid in the different formulations administered to Wistar rats.

Formulation	CC (%)	DMC (%)	BDMC (%)	Total (%)
NPZ-CC	7.29	1.41	0.48	9.18
Curarti^®^	14.83	2.85	0.86	18.54
CPC	15.71	3.81	0.48	20.00
Oral Curcuminoid extract	71.71	16.16	6.37	94.24

**Table 6:** Pharmacokinetic variables of peak concentration (Cmax; ng/mL), time at which the Cmax is reached (Tmax; h), rea under the plasma concentration–time curve from time 0 to last observation (AUC_0-tlast_; ng·h/mL), and relative oral bioavailability (Fr) for the three curcuminoids per formulation. Statistical differences between NPZ-CC, Curarti^®^, and CPC formulations vs. oral curcuminoids are marked with asterisks: *p* < 0.05 (*), *p* < 0.01 (**) and *p* < 0.001 (***). Data are expressed as mean ± SEM.

Compound	Tmax (h)	Cmax (ng/mL)	AUC_0-tlast_ (ng·h^−1^·mL^−1^)	Fr ^2^
*CC*				
Free extract	6.00 ± 0.00	186.29 ± 76.54	2438.74 ± 944.76	-
Curarti^®^	0.50 ± 0.13 ***	2707.94 ± 2148.35	7698.28 ± 2994.08	2.98 ± 0.15
CPC	0.25 ± 2.88	1245.46 ± 410.51 *	15,458.74 ± 2276.17	6.09 ± 0.29
NPZ-CC	0.25 ± 0.63 ***	1518.81 ± 118.27 *	19,260.28 ± 3828.87	7.51 ± 0.39
*DMC*				
Free extract	4.00 ± 1.75	17.21 ± 0.13	208.93 ± 10.66	-
Curarti^®^	0.50 ± 0.50	231.53 ± 166.03	632.23 ± 181.59	3.35 ± 0.09
CPC	0.25 ± 0.38	789.58 ± 521.08	3163.82 ± 635.17 *	13.52 ± 0.31
NPZ-CC	0.25 ± 0.00	112.08 ± 4.47 **	1109.18 ± 221.92	5.80 ± 0.11
*BDMC*				
Free extract	1.00 ± 0.00	0.48 ± 0.13	1.10 ± 0.47	-
Curarti^®^	0.50 ± 0.13	8.31 ± 5.42	15.07 ± 7.04	19.79 ± 1.24
CPC	0.25 ± 0.00	56.44 ± 24.52	214.57 ± 105.29	548.53 ± 1.99
NPZ-CC	0.25 ± 0.00	112.08 ± 4.47 **	188.27 ± 110.27	219.31 ± 13.01
Total curcuminoids ^1^				
Free extract	0.50 ± 0.00	186.29 ± 62.50	2209.64 ± 1275.54	-
Curarti^®^	0.50 ± 0.4	2947.78 ± 1359.18	10,259.82 ± 1806.52	4.64 ± 0.34
CPC	0.25 ± 2.34	2274.97 ± 911.74	18,893.16 ± 4449.39	8.55 ± 0.69
NPZ-CC	0.25 ± 0.5	1742.97 ± 109.46	20,265.71 ± 3733.05	9.17 ± 0.63

^1^ Total curcuminoid pharmacokinetic metrics should be handled with cautious, as DMC and BDMC kinetics seem to involve non-linear processes or saturable mechanisms according to observations by Cuomo, et al. [46]. ^2^ Pairwise t-test results for comparison of relative bioavailability (only detected significant differences based on p-values are provided). For CC, *p*-values < 0.001 when comparing NPZ-CC vs. Curarti^®^, NPZ-CC vs. CPC, and Curarti^®^ vs. CPC. For DMC, *p*-values < 0.001 when comparing NPZ-CC vs. Curarti^®^, NPZ-CC vs. CPC, and Curarti^®^ vs. CPC. For BDMC, *p*-values < 0.001 when comparing NPZ-CC vs. Curarti^®^ and Curarti^®^ vs. CPC. For total curcuminoids, *p*-values < 0.001 when comparing NPZ-CC vs. Curarti^®^ and Curarti^®^ vs. CPC.

## Data Availability

Data will be made available on a reasonable request.
